# Mediterranean Diet Adherence and Health-Related Quality of Life during Pregnancy: Is the Mediterranean Diet Beneficial in Non-Mediterranean Countries?

**DOI:** 10.3390/nu16050718

**Published:** 2024-03-01

**Authors:** Marta Flor-Alemany, Johanna Sandborg, Jairo H. Migueles, Emmie Söderström, Maria Henström, Nuria Marín-Jiménez, Laura Baena-García, Virginia A. Aparicio, Marie Löf

**Affiliations:** 1 Department of Physiology, University of Granada, 18071 Granada, Spain; virginiaparicio@ugr.es; 2 Biomedical Research Centre (CIBM), Institute of Nutrition and Food Technology (INYTA), University of Granada, 18016 Granada, Spain; 3 Sport and Health University Research Institute (IMUDS), 18007 Granada, Spain; nuriaproyecto@gmail.com (N.M.-J.); lbaenagarcia@ugr.es (L.B.-G.); 4 Department of Biosciences and Nutrition, Karolinska Institutet, 14152 Huddinge, Sweden; johanna.sandborg@ki.se (J.S.); maria.henstrom@ki.se (M.H.); marie.lof@ki.se (M.L.); 5 PROFITH “Promoting FITness and Health through Physical Activity” Research Group, Department of Physical Education and Sports, Faculty of Sport Sciences, University of Granada, 18071 Granada, Spain; jairo.hidalgo.migueles@gmail.com; 6 Department of Health, Medicine and Caring Sciences, Linköping University, 58183 Linköping, Sweden; emmie.soderstrom@liu.se; 7 GALENO Research Group, Department of Physical Education, Faculty of Education Sciences, University of Cádiz, 11519 Cadiz, Spain; 8 Department of Nursing, Faculty of Health Sciences, University of Granada, 18016 Granada, Spain; 9 Instituto de Investigación Biosanitaria (IBS), 18012 Granada, Spain

**Keywords:** diet, Mediterranean, mental health, pregnancy, pregnant women, Short-Form Health Survey 36

## Abstract

This study aimed to examine the association of Mediterranean diet (MD) adherence and MD components with health-related quality of life (HRQoL) in pregnant women from Spain and Sweden. A total of 138 pregnant women from Spain (age: 32.9 ± 4.6 years old) and 302 pregnant women from Sweden (age: 31.3 ± 4.1 years old) were included. MD adherence was assessed with the Mediterranean food pattern (i.e., a MD index) at the 14–16th gestational weeks. HRQoL was assessed with the Spanish and Swedish versions of the 36-item Short-Form Health Survey (SF-36 and RAND-36, respectively) at the 14–16th and 34–37th gestational weeks. A greater MD adherence was associated with better physical functioning, bodily pain, vitality, emotional role, and mental health in cross-sectional associations (2nd trimester) in the Spanish sample (all *p* < 0.05). Furthermore, a greater MD adherence was associated with lower bodily pain in both Spanish and Swedish samples (both *p* < 0.05) in the 3rd trimester. The associations of MD adherence with pain seem to be explained by a greater intake of fiber, fish, fruits, nuts, and legumes (all *p* < 0.05). A greater MD adherence, driven by a higher intake of fiber, fish, fruits, nuts, and legumes, was associated with lower pain throughout pregnancy in both Mediterranean and non-Mediterranean populations.

## 1. Introduction

Health-related quality of life (HRQoL) is a concept determining the self-perception of health-related physiological, psychological, and social constructs [1]. Previous evidence showed that pregnant women may experience a decrease in their HRQoL throughout pregnancy [2,3]. This HRQoL reduction might have clinical consequences as it is associated with both maternal and infant health (e.g., maternal postpartum mental health and the psychomotor development of the infant) [4,5].

The Mediterranean diet (MD) is a traditional dietary pattern historically prevalent in Mediterranean regions of Southern Europe, characterized by a substantial consumption of vegetables, legumes, fruits, nuts, cereals, and olive oil, along with a low intake of saturated fats, a moderate consumption of fish, and limited quantities of dairy products, meat, and poultry [6]. The MD has been recognized as one of the healthiest dietary patterns in Europe [6]. Adherence to the MD has been linked to enhanced health-related quality of life (HRQoL) among non-pregnant adults, both by observational [7,8,9,10] and intervention studies [11]. However, the correlation between adherence to the MD and its specific components (e.g., fruits, vegetables, whole grains, and fish) and HRQoL during pregnancy has yet to be explored. This inquiry is particularly significant considering the observed departure of pregnant women from the Mediterranean dietary pattern [12,13], even in Mediterranean regions such as Spain.

Most of the research on the benefits of a MD has been conducted in Mediterranean countries (e.g., Spain, Italy, and Greece). Yet its benefits in non-Mediterranean countries, if confirmed, might help inform policymakers to develop policies to promote the MD worldwide. The studies examining the benefits of a MD in non-Mediterranean countries showed that adhering to a MD was associated with lower cardiovascular disease incidence and mortality in the UK [14], lower mortality rates among Australian adults [15], as well as lower risk for Parkinson’s disease and depression in Swedish middle-aged women [16,17]. However, there are a limited number of studies directly comparing the effects of the Mediterranean diet in different contexts, i.e., non-Mediterranean countries versus Mediterranean countries. Variations in cultures and societies make it difficult to compare the MD indices across countries and, thus, to compare the benefits of this dietary pattern. Investigating the same index, used consistently in a Mediterranean and a non-Mediterranean region, would provide internal consistency to shed light on that hypothesis. Additionally, MD benefits go beyond cardiovascular health, and they could be especially useful in specific periods of life, such as pregnancy, as this period is characterized by substantial physiological and psychological changes. Therefore, we aimed to investigate the associations of MD adherence with HRQoL in a Mediterranean population (i.e., pregnant women from Spain) and in a non-Mediterranean population of pregnant women (i.e., pregnant women from Sweden), assessing MD adherence in a consistent manner. The findings of this study could provide valuable insights into the potential benefits of following a MD during pregnancy, even in different cultural and geographical contexts.

## 2. Materials and Methods

### 2.1. Study Design and Participants

This study included data from two projects: the GESTAtion and FITness project from Spain (GESTAFIT, hereinafter Study 1) and the HealthyMoms trial from Sweden (hereinafter Study 2).

#### 2.1.1. Study 1

These are secondary analyses from the GESTAFIT project, where a concurrent exercise intervention (i.e., aerobic plus strength training) from the 17th gestational week (g.w.) until birth was conducted [18]. Participants were recruited during the first gynecologist routine visit (at the 12th g.w.) at “San Cecilio” and “Vigen de las Nieves” University Hospitals in Granada, Spain. A total of 159 Spanish pregnant women met the inclusion–exclusion criteria (Supplementary Table S1) and signed a written informed consent. Among them, a total of 138 women who had valid data on sociodemographic characteristics, MD adherence, and HRQoL at the 16th g.w. were included in the present study.

#### 2.1.2. Study 2

These are secondary analyses from the HealthyMoms mHealth-based randomized controlled trial [19,20]. In short, the intervention group used the HealthyMoms smartphone application in addition to standard care. Participants were recruited for this study during the first routine visit to the maternity clinics in Linköping, Norrköping, and Motala in Sweden. A total of 305 Swedish women met the inclusion–exclusion criteria (Supplementary Table S1) and were included in this study. Among them, a total of 302 had valid data on MD adherence and HRQoL at the 14th g.w. and were included in the present study.

### 2.2. Sociodemographic Characteristics

#### Study 1 and 2

Pregnant women reported demographic and clinical information via questionnaires. The women answered questions about their age, educational level, working status, weight before pregnancy, and number of previous pregnancies lasting more than 30 weeks. Upon entering this study, their height and weight were measured. Pre-pregnancy body mass index (BMI) was calculated as reported weight (kg) prior to pregnancy divided by measured height (m) at the 14–16th g.w. squared (kg/m^2^).

### 2.3. Health-Related Quality of Life

#### Study 1 and 2

The HRQoL of the participants was assessed using the Spanish [21] and Swedish [22] versions of the Short-Form Health Survey 36 (SF-36). Scale scores for RAND-36 and SF-36 are identical for six of the eight scales, whereas scoring algorithms for the pain and general health scales are slightly different. However, Hays et al. demonstrated that the correlation between the scales using the two different scoring algorithms was 0.99 in the MOS study, indicating that the difference is negligible [23]. The SF-36 has been widely employed in pregnant women [1,2,4,24,25] and comprises 36 items that are grouped into eight domains: physical functioning, physical role, bodily pain, general health, vitality, social functioning, emotional role, and mental health. The number of items varies from two to ten between dimensions. The items are multiple-choice, with two to six answer options, that are subsequently recategorized to be summed and transformed into scales ranging from 0 (the worst possible health state) to 100 (the best possible state). This accounts for all the subscales, with the pain score being reversed, where a higher score indicates lower pain.

### 2.4. Dietary Assessment

#### 2.4.1. Study 1

Dietary habits were assessed at the 16th and 34th g.w. with a food frequency questionnaire validated for the Spanish non-pregnant adult population [26]. Women were asked about the frequency of consumption of the different food groups (never or number of times per day, week, month, or year).

#### 2.4.2. Study 2

Dietary habits were assessed at the 14th and 37th g.w. with the web-based dietary recall method Riksmaten FLEX, developed by the Swedish National Food Agency and adapted for pregnant women [27]. Full details of the data collection and processing have been provided previously [20]. Briefly, the method utilized 3-day, 24 h recalls [27], and the reported intakes of foods and drinks were averaged per day. Participants were instructed to report their dietary consumption for that day and the day before at their first log-in. To ensure that registrations comprised both weekdays and weekend days, the third day was automatically assigned to occur within 7 days of the first registration, on either a weekday or a weekend day, depending on the day of the first registration [27].

### 2.5. Mediterranean Diet Adherence

#### Study 1 and 2

With the data obtained from the food frequency questionnaire (Study 1) and the 3-day, 24 h recalls (Study 2), the Mediterranean food pattern [28] (i.e., a MD adherence index) was constructed as previously described [29]. The Mediterranean food pattern is a quintile-based sum score of eight food groups (olive oil, fiber, fruits, vegetables, fish, cereals, meat, and alcohol). The total score ranges from 5 to 40. In the present study, alcohol consumption was not considered because the women were pregnant. Therefore, the maximum score ranges from 4 to 35, where higher scores indicate greater MD adherence. Participants were classified as having high MD adherence if they had a score of ≥21 points in the MD score, as previously classified in the Study 1 sample [30].

### 2.6. Statistical Analysis

#### Study 1 and 2

Descriptive statistics were summarized as mean (standard deviation) for quantitative variables and frequency (%) for categorical variables, as appropriate. Differences in HRQoL throughout pregnancy (i.e., from the 14–16th to the 34–37th g.w.) were compared by the paired Student’s *t*-test. Associations between MD adherence, food group intake, and HRQoL were evaluated through multiple linear regression analyses after adjusting for age (years), parity (primipara or multipara), and educational level (high school or less or university degree). We performed sensitivity analyses, additionally adjusting for physical activity levels given their potential effect on HRQOL; however, the results remained the same. Longitudinal analyses were additionally adjusted by baseline values (14–16th g.w.), MD adherence changes (i.e., MD adherence at the 34–37th gestational week minus MD adherence at the 14–16th gestational week), and group allocation (i.e., control or intervention). All analyses were conducted using the Statistical Package for Social Sciences (IBM SPSS Statistics for Windows, version 22.0, Armonk, NY, USA), and the level of significance was set at *p* ≤ 0.05.

## 3. Results

The clinical and sociodemographic characteristics of study participants are presented in Table 1.

Lower scores in physical functioning, physical role, and bodily pain were observed in the third trimester compared to the second trimester in both Spanish and Swedish populations (all *p* < 0.05) (Table 2). Furthermore, the Swedish sample improved energy/fatigue and role-functioning/emotional scores in the 14th to 37th gestational weeks (both *p* < 0.05).

Cross-sectional associations of MD adherence with HRQoL in the 2nd trimester (14–16th g.w.) are shown in Table 3. In the Spanish sample (Study 1), a greater MD adherence was associated with better SF-36 physical functioning, vitality, emotional role, and mental health (β ranging from 0.176 to 0.190, all *p* < 0.05), whereas no associations were found between MD adherence and HRQoL in the Swedish sample.

Longitudinal associations of MD adherence in the 2nd trimester with HRQoL in the 3rd trimester are shown in Table 4. A greater MD adherence was associated with lower bodily pain in both samples (β ranging from 0.186 to 0.215, both *p* < 0.05). Otherwise, MD adherence was not longitudinally associated with any of the other HRQoL components in any of the study samples (Table 4).

Longitudinal associations of individual food groups with the pain dimension are shown in Table 5. A greater intake of fiber and fruits was associated with lower bodily pain in both Spanish and Swedish populations (β ranging from 0.172 to 0.252, all *p* < 0.05). Furthermore, in the Spanish sample, a greater intake of fish was associated with lower bodily pain (β = 0.228, *p* = 0.020). In the Swedish sample, a greater intake of nuts and legumes was associated with lower bodily pain (β = 0.165, *p* = 0.011, and β = 0.155, *p* = 0.049, respectively).

## 4. Discussion

Our results suggest that a greater MD adherence during pregnancy was associated with lower bodily pain in both Mediterranean and non-Mediterranean populations. These associations seem to be driven by a higher intake of fiber, nuts, legumes, fish, and fruits.

HRQoL is a conceptualization reflecting an individual’s physical and mental well-being and has emerged as an essential consideration in the prevention and treatment of diseases [31]. HRQoL deteriorates in women over the course of pregnancy [2,24,32]. This study found that physical functioning, physical role, and bodily pain worsened throughout gestation (Study 1), whereas energy/fatigue and role-functioning/emotional scores improved (Study 2), which is highly in agreement with previous evidence reporting that physical domains decline [2,24] and mental domains improve as pregnancy progresses [24].

Previous literature [33] has reported an association between MD adherence and HRQoL, suggesting that this dietary pattern may be beneficial for HRQoL in the non-pregnant adult population, whereas others did not observe such an association [34]. This study sets a promising area of research as it seems that adhering to a MD during pregnancy may improve an important physical symptom of the pregnancy, bodily pain. This observation is corroborated with data from two studies conducted in different settings, the GESTAFIT study in Spain and the HealthyMoms trial in Sweden, thus involving a Mediterranean and a non-Mediterranean region. Further research should confirm this observation, and trials should investigate the hypothesis of whether greater MD adherence during pregnancy actually reduces bodily pain symptoms by the end of the pregnancy, as well as extend the question to other physical symptoms.

Apart from the MD, there are indeed other studies that have investigated the adherence to other dietary patterns in relation to pregnancy health outcomes. A study conducted by Miura et al. [35] showed that following a “Japanese” dietary pattern, characterized by the intake of foods such as rice, miso soup, Japanese wheat noodles, cereals, beans, vegetables, fruits, meats, Japanese tea, vegetable juice, and 100% fruit juice, and also by a low intake of milk, was associated with poor mental and physical HRQoL in pregnant women in the first trimester of gestation. Moreover, a medium–high intake of an “unbalanced” dietary pattern, characterized by a high intake of grain cereals, bottled tea, bottled coffee, and carbonated drinks, and a low intake of green and yellow vegetables, fruits, and green tea, was associated with poorer physical and mental HRQoL. Furthermore, a study that included 144 pregnant women in the first trimester of gestation showed that proper nutritional habits that take into consideration the type of food consumed (wholegrain bread, vegetables, or fruit) were strongly correlated with all aspects of HRQoL in pregnant women with high General Index of Intensity of Health Behaviours [36]. Nonetheless, neither diet quality nor dietary patterns were considered [36]. However, in these previous studies [35,36], HRQoL was only assessed during the first trimester of pregnancy. In the present study, we explored the associations between MD adherence and HRQoL from the early 2nd to the late 3rd trimester, gaining insights on the associations of a healthy dietary pattern (in this case, the MD) with HRQoL along the pregnancy course. Moreover, we also studied the different food groups that comprise the Mediterranean dietary pattern, finding that a higher intake of fiber, fish, fruits, nuts, and legumes was associated with better HRQoL during pregnancy. Our findings point out the promotion of the intake of fruit, fiber, fish, and nuts during pregnancy to produce an improvement in bodily pain at the end of the pregnancy.

One of the most accredited hypotheses is that a MD is positively associated with better overall health status and a reduced risk of major chronic diseases because of its high content of different beneficial compounds, such as antioxidants (largely present in leafy vegetables and fruits), dietary fiber, polyunsatured fatty acids (mainly from fish and nuts), and monounsatured fatty acids (olive oil) [33,37]. A recent study has offered interesting indications on this issue by highlighting a major role played by dietary antioxidants in explaining the relationship between a MD and HRQoL [33]. Epidemiological studies have confirmed a correlation between diet and depression, pointing out the importance of a diet rich in antioxidants and other essential compounds typical of a MD in reducing the risk of depression, which may explain its positive effects in improving mental health [38]. Regarding physical health and pain, there are several biological and physiological mechanisms that could explain the beneficial effects of a MD pattern, such as reduced low-grade chronic inflammation, coagulation markers, and improved endothelial function [39,40,41]. In fact, several foods or substances with functional properties have been studied for their anti-inflammatory effects and/or their possible treatment of pain, such as omega-3 present in fish oil [42] and several flavonoids in fruits [43]. Nociceptors are activated by inflammatory mediators; however, when they are constantly exposed to these mediators (i.e., a state of chronic inflammation), peripheral and central sensitization occur, leading to pain chronification [41]. In this regard, our findings about pain are in agreement with the current literature that attributes the Mediterranean diet as having anti-inflammatory and analgesic effects [40].

Cross-sectionally, we found differences in the associations of MD adherence with HRQoL in the Spanish and Swedish samples. Differences in context, nutritional habits, public health systems, and populations make it impossible to draw a coherent explanation of what might be driving these different associations. However, when looking at a similar period of life, pregnancy, it was interesting that the associations of MD adherence with pain were consistent in both populations. Therefore, a MD may attenuate the experience of bodily pain, especially in the third trimester of pregnancy, when women generally experience a deterioration in HRQoL. As a result, the benefits of MD adherence might be transferable to non-Mediterranean countries.

Some limitations need to be highlighted. Firstly, given the observational design of the present study, a clear cause–effect identification is not possible. As a result, we cannot determine whether a healthier diet affects HRQoL or, on the contrary, whether a poorer HRQoL leads to unhealthy dietary behaviors. Secondly, the participants were enrolled in an exercise intervention (Study 1) and an mHealth-based intervention (Study 2) that might affect our findings regarding the 34–37th g.w. However, we included group allocation as a confounder in our longitudinal analyses to account for the possible effect of the intervention conducted within the GESTAFIT and Healthymoms projects on these outcomes. The two distinct studies compared in this manuscript employed different methods for collecting dietary data. Study 1 utilized a FFQ, while Study 2 utilized three 24 h dietary recalls. Discrepancies between the two studies may arise from the differing types of data collection methods. Additionally, the study sample might not be representative of the Spanish or Swedish pregnant populations; therefore, the external validity of our findings should be investigated in future studies. There were no diagnosed comorbidities that might drive the associations with the bodily pain domain. Nonetheless, residual confounding might still be driving part of the associations, as it may occur in any observational design. Our associations seem to exhibit a statistically small effect size, approximately 0.2 standard deviations. Nonetheless, it remains uncertain whether this magnitude of effect translates into clinical relevance, which warrants further investigation in future studies. Several strengths of this study are worth considering. A detailed definition of the intake of foods and drinks and a valid assessment of MD adherence were employed. Furthermore, the measurement tool employed to assess HRQoL, despite not being a pregnancy-specific tool, is widely valid and reliable [44]. Additionally, HRQoL was assessed at multiple time points (i.e., 2nd and 3rd trimesters) and assessed using valid and reliable dietary assessment methods, which guarantees the quality of the index.

## 5. Conclusions

A greater MD adherence was associated with lower pain throughout gestation in both Mediterranean and non-Mediterranean populations. Specifically, a greater intake of fiber, fish, fruits, nuts, and legumes seemed to explain these associations. Our findings may emphasize the importance of improving MD adherence during pregnancy in future community and population-based programs or policies to improve HRQoL throughout pregnancy, considering both Mediterranean and non-Mediterranean contexts. Given the limited number of studies available, further research is warranted to explore the impact of maternal healthy dietary habits on HRQoL during pregnancy and investigate causality and its mechanisms. These findings could support the implementation of nutritional interventions during pregnancy that promote greater adherence to the MD and ultimately enhance maternal HRQoL and fetal well-being.

## Figures and Tables

**Table 1:** Characteristics of the study samples at baseline (14–16th gestational week).

	Study 1		Study 2	
	*N*	Mean (SD)	*N*	Mean (SD)
Age at baseline (years)	138	32.9 (4.6)	302	31.3 (4.1)
Pre-pregnancy BMI (kg/m^2^)	125	24.1 (4.3)	302	23.9 (3.8)
Pre-pregnancy BMI categories (*n*, %)	*N*	%	*N*	%
Underweight (<18.5 kg/m^2^)	4	3.2	6	2.0
Normalweight (18.5–24.9 kg/m^2^)	78	62.4	209	69.2
Overweight (25.0–29.9 kg/m^2^)	29	23.2	67	22.2
Obesity (>30 kg/m^2^)	14	11.2	20	6.6
Mediterranean diet adherence (14–16th g.w.)				
High Mediterranean diet (*n*, %)	57	41.3	57	18.9
Mediterranean diet adherence (34–37th g.w.)				
High Mediterranean diet (*n*, %)	52	47.7	59	22.1
Parity (*n* %)				
Primipara	81	58.7	175	57.9
Multipara	57	41.3	127	42.1
Level of education (*n* %)				
High school or less	17	12.3	68	22.5
University degree	121	87.7	234	77.5

Descriptive statistics were summarized as mean (standard deviation) for quantitative variables and frequency (%) for categorical variables, as appropriate. BMI, body mass index; G.W., gestational week.

**Table 2:** Differences in health-related quality of life by gestational week.

Study 1 (*n* = 109)	16th Gestational Week	34th Gestational Week	*p*
Health-related quality of life			
Physical functioning (0–100)	83.0 (13.7)	67.7 (19.2)	<0.001
Physical role (0–100)	67.7 (24.1)	55.1 (23.7)	<0.001
Bodily pain (0–100)	61.5 (25.2)	53.4 (26.3)	0.001
General health (0–100)	77.3 (15.7)	78.1 (18.1)	0.532
Vitality (0–100)	53.7 (17.2)	52.4 (17.5)	0.408
Social functioning (0–100)	78.8 (21.9)	76.5 (21.8)	0.327
Emotional role (0–100)	91.0 (14.6)	91.1 (15.5)	0.569
Mental health (0–100)	75.7 (14.0)	74.5 (15.8)	0.393
**Study 2 (*n* = 273)**	**14th Gestational Week**	**37th Gestational Week**	***p***
Health-related quality of life			
Physical functioning	90.4 (10.9)	59.2 (20.1)	<0.001
Role-functioning/physical	68.9 (34.7)	33.8 (35.4)	<0.001
Pain	85.6 (17.5)	64.1 (22.5)	<0.001
General health	77.9 (16.1)	78.6 (15.9)	0.389
Energy/fatigue	48.2 (17.8)	51.5 (15.6)	0.002
Social functioning	84.1 (19.3)	83.6 (19.5)	0.771
Role-functioning/emotional	85.4 (29.7)	89.4 (25.8)	0.047
Emotional well-being	79.7 (12.9)	81.1 (11.2)	0.052

Values shown as mean (standard deviation). *p* values derived from paired Student’s *t*-test.

**Table 3:** Cross-sectional associations of Mediterranean diet adherence with health-related quality of life.

	2nd Trimester (Study 1) (*n* = 138)		2nd Trimester (Study 2) (*n* = 302)	
HRQoL	Β	*p*	β	*p*
Physical functioning (0–100)	0.183	0.038	0.085	0.148
Physical role (0–100)	0.108	0.229	−0.070	0.233
Bodily pain (0–100)	0.136	0.116	0.006	0.924
General health (0–100)	0.153	0.088	0.095	0.108
Vitality (0–100)	0.190	0.031	−0.007	0.908
Social functioning (0–100)	0.119	0.188	0.032	0.582
Emotional role (0–100)	0.191	0.032	−0.073	0.220
Mental health (0–100)	0.176	0.049	−0.015	0.804

*p* values derived from multiple linear regression adjusted by age, parity, and educational level. HRQoL, health-related quality of life.

**Table 4:** Longitudinal associations of Mediterranean diet adherence with health-related quality of life.

	Diet at the 2nd and HRQoL at the 3rd Trimester (Study 1) (*n* = 105)		Diet at the 2nd and HRQoL at the 3rd Trimester (Study 2) (*n* = 263)	
HRQoL	β	*p*	β	*p*
Physical functioning (0–100)	0.060	0.460	0.115	0.089
Physical role (0–100)	0.043	0.671	0.038	0.583
Bodily pain (0–100)	0.215	0.016	0.186	0.005
General health (0–100)	0.046	0.567	0.030	0.584
Vitality (0–100)	0.027	0.781	0.106	0.087
Social functioning (0–100)	0.167	0.084	0.022	0.750
Emotional role (0–100)	0.042	0.664	0.066	0.328
Mental health (0–100)	−0.005	0.956	0.004	0.949

*p* values derived from multiple linear regression adjusted by age, parity, educational level, Mediterranean diet adherence changes, baseline health-related quality of life, and group allocation.

**Table 5:** Longitudinal associations of food groups with pain throughout pregnancy.

	Diet at the 2nd and HRQoL at the 3rd Trimester (Study 1) (*n* = 105)		Diet at the 2nd and HRQoL at the 3rd Trimester (Study 2) (*n* = 263)	
Pain	Β	*p*	β	*p*
High glycemic foods (g/day)	−0.092	0.335	−0.094	0.228
Olive oil (g/day)	0.250	0.070	−0.054	0.409
Fiber (g/day)	0.172	0.042	0.173	0.014
Fish (g/day)	0.228	0.020	0.147	0.082
Fruits (g/day)	0.252	0.006	0.202	0.003
Nuts (g/day)	0.202	0.118	0.165	0.011
Vegetables (g/day)	0.039	0.655	0.073	0.316
Legumes (g/day)	0.177	0.165	0.155	0.049
Meat and subproducts (g/day)	−0.047	0.597	−0.057	0.425

*p* values derived from multiple linear regression adjusted by age, parity, educational level, Mediterranean diet adherence changes, baseline health-related quality of life, and group allocation.

## Data Availability

The datasets used and/or analyzed during the current study are available from the corresponding author on reasonable request. The data are not publicly available due to their containing information that could compromise the privacy of research participants.

## Supplementary Materials

The following supporting information can be downloaded at: https://www.mdpi.com/article/10.3390/nu16050718/s1, Table S1: Inclusion and exclusion criteria in the GESTAFIT and Healthymoms projects.
