# Supplementary material for: Mediterranean Diet Adherence and Health-Related Quality of Life during Pregnancy: Is the Mediterranean Diet Beneficial in Non-Mediterranean Countries?
Source: Nutrients. 2024 Mar 1;16(5):718. doi: 10.3390/nu16050718 (PMC10933844; doi:10.3390/nu16050718)
Supplement: Supplementary file 1 [file nutrients-16-00718-s001.zip › nutrients-2878386-supplementary.pdf]

Supplementary Material

Supplement to: "**Mediterranean diet adherence and health-related quality of life during pregnancy: is the Mediterranean diet beneficial in non-Mediterranean countries?".**

Table of contents

Supplementary Table S1. Inclusion and exclusion criteria in the GESTAFIT and Healthymoms project.

Supplementary Table S1. Inclusion and exclusion criteria in the GESTAFIT and Healthymoms project.

**GESTAFIT Project**
**Inclusion criteria**
- Pregnant women aged 25-40 years old with a normal pregnancy course.<br>- Answering "no" to all questions on the PARmed-X for pregnancy.<br>- Being able to walk without assistance.<br>- Being able to read and write properly.<br>- Informed consent: Being capable and willing to provide written consent.
**Exclusion criteria**
- Having acute or terminal illness.<br>- Having malnutrition.<br>· Being unable to conduct tests for assessing physical fitness or exercise during pregnancy.<br>- Having pregnancy risk factors (such as hypertension, type 2 diabetes, etc.).<br>- Having a multiple pregnancy.<br>- Having chromosopathy or foetal malformations.<br>- Having uterine growth restriction.<br>- Having foetal death.<br>- Having upper or lower extremity fracture in the past 3 months.<br>- Suffering neuromuscular disease or presence of drugs affecting neuromuscular function.<br>- Being registered in another exercise program.<br>- Performing more than 300 minutes of at least moderate physical activity per week.<br>-Being engaged in another physical exercise program<br>- Being unwilling either to complete the study requirements or to be randomized into the control or intervention group.
**Healthymoms Project**
**Inclusion criteria**
-Pregnant women aged 18 years or older<br>-Singleton pregnancy<br>-Ability to read and speak the Swedish language good enough to understand the HealthyMoms app and to provide informed consent
**Exclusion criteria**
-History of an eating disorder<br>-Pre-pregnancy diabetes, and other medical conditions or pharmacological treatment that could alter body weight prior to gestation
